# Detection of *BRAF* mutations in the tumour and serum of patients enrolled in the AZD6244 (ARRY-142886) advanced melanoma phase II study

**DOI:** 10.1038/sj.bjc.6605371

**Published:** 2009-10-27

**Authors:** R E Board, G Ellison, M C M Orr, K R Kemsley, G McWalter, L Y Blockley, S P Dearden, C Morris, M Ranson, M V Cantarini, C Dive, A Hughes

**Affiliations:** 1 AstraZeneca Pharmaceuticals, Alderley Park, Cheshire SK10 4TG, UK; 2 Clinical and Experimental Pharmacology Group, Paterson Institute for Cancer Research, University of Manchester M20 4BX, UK; 3 CRUK Department of Medical Oncology, Christie Hospital NHS Trust, Wilmslow Road, Manchester M20 4BX, UK

**Keywords:** AZD6244, amplification refractory mutation system, *BRAF*, circulating free DNA, cutaneous melanoma

## Abstract

**Background:**

This study investigated the potential clinical utility of circulating free DNA (cfDNA) as a source of *BRAF* mutation detection in patients enrolled into a phase II study of AZD6244, a specific MEK1/2 inhibitor, in patients with advanced melanoma.

**Methods:**

*BRAF* mutations were detected using Amplification Refractory Mutation System allele-specific PCR. *BRAF* mutation status was assessed in serum-derived cfDNA from 126 patients enrolled into the study and from 94 matched tumour samples.

**Results:**

Of 94 tumour samples, 45 (47.9%) were found to be *BRAF* mutation positive (*BRAF*+). Serum-derived cfDNA was *BRAF*+ in 33 of 126 (26.2%) samples, including in five samples for which tumour data were unavailable. Of *BRAF*+ tumours, 25 of 45 (55.6%) were *BRAF*+ in cfDNA. In three cases in which the tumour was negative, cfDNA was *BRAF*+. Progression-free survival (PFS) of patients with *BRAF*+ tumour and cfDNA was not significantly different compared with tumour *BRAF*+ but cfDNA *BRAF-*negative patients, indicating that cfDNA *BRAF* detection is not associated with poorer prognosis on PFS in stage III/IV advanced melanoma.

**Conclusions:**

These data demonstrate the feasibility of *BRAF* mutation detection in cfDNA of patients with advanced melanoma. Future studies should aim to incorporate *BRAF* mutation testing in cfDNA to further validate this biomarker for patient selection.

Somatic mutations in the *BRAF* oncogene have been documented at a high frequency in cutaneous melanomas, occurring in up to 60% of cell lines and tumour samples (Brose *et al*, 2002; Davies *et al*, 2002). The most documented *BRAF* mutation is a thymidine-to-adenine switch at nucleotide position 1799 in exon 15 (Davies *et al*, 2002). This encodes for a valine-to-glutamic acid substitution at amino acid 600 in the kinase domain of the protein (p.V600E, previously known as p.V599E; Smalley and Herlyn, 2004). The resulting structural change mimics phosphorylated BRAF and has an elevated basal kinase activity substantially higher than that for wild-type *BRAF*, causing constitutive activation of downstream signalling in the Ras–Raf–MEK–extracellular ligand-regulated kinase pathway, which can lead to malignant transformation (Mansour *et al*, 1994).

AZD6244 is an orally available, potent, selective, ATP-uncompetitive inhibitor of MEK1/2, with pre-clinical activity in *BRAF* mutation-positive (*BRAF*+) and *RAS* mutation-positive tumour models (Yeh *et al*, 2007). Clinical responses were observed in a recent 200-patient phase II study of AZD6244 *vs* temozolomide in patients with advanced melanoma (study D1532C00003, Figure 1; Dummer *et al*, 2008). Six patients randomised to AZD6244 had a confirmed partial response, of whom five had *BRAF*+ tumours. Nine patients randomised to temozolomide had a confirmed partial response, three of whom had *BRAF*+ tumours. In subsequent clinical trials of this drug, patients will be screened and selected on the basis of the mutational status of their tumour. In particular, future clinical trials of AZD6244 in advanced melanoma will evaluate patient selection on the basis of tumour mutation status.

Mutation status is traditionally assessed by analysis of DNA extracted from archival tumour tissue samples. However, biopsy material is not always readily available, even in patients with metastatic melanoma. Furthermore, limited and degraded amounts of DNA extracted from tumour biopsies and formalin-fixed paraffin-embedded (FFPE) tissues present an inherent technical challenge in mutation detection. If the response to a therapeutic agent depends on the presence or absence of a particular DNA mutation, then the availability of tumour-derived DNA for mutation analysis becomes critically important.

An alternative source of tumour-derived DNA is cell-free or circulating free DNA (cfDNA). This can be extracted from plasma and serum, providing an opportunity to develop a less-invasive and more accessible source of tumour DNA for mutation detection. Previous studies have demonstrated the feasibility of detecting tumour-specific mutations in cfDNA from patients with cancer, including detection of epidermal growth factor receptor mutations in patients with non-small-cell lung cancer (Kimura *et al*, 2006, 2007) and *KRAS* mutations in patients with pancreatic and colorectal cancers (Sorenson, 2000). More recently, *BRAF* mutations have been detected in cfDNA of patients with melanoma (Daniotti *et al*, 2007; Shinozaki *et al*, 2007; Yancovitz *et al*, 2007). However, these initial studies included a small number of patients with only limited numbers of matched tumour samples.

In this study, we investigated the potential utility of cfDNA for the detection of *BRAF* mutation status in a large group of patients enrolled into the AZD6244 advanced melanoma phase II study to determine whether cfDNA mutations could be used for patient selection as an alternative to tissue biopsies.

## Materials and methods

### Patients and samples

A total of 200 patients with advanced melanoma were enrolled into study D1532C00003. The study was conducted according to Good Clinical Practice and the Declaration of Helsinki. All patients provided written informed consent before participation in the main study. Consent for analysis of tumour biopsy material was obtained from all patients enrolled in the study and additional voluntary consent for collection of serum samples for genetic analysis was given by 126 patients.

### Blood processing for cfDNA extraction

At the time of enrolment, 8.5 ml of peripheral blood was taken in a Becton-Dickinson Vacutainer Serum Collection Tube and gently inverted for a minimum of five times to ensure a thorough mixing of the sample. The blood was allowed to clot for 30 min and was centrifuged at 2000 g for 10 min. The resultant serum supernatant was transferred to a clean tube and stored at −80°C until analysis.

For cfDNA extraction, serum was thawed at room temperature and cfDNA was extracted from 1 ml of serum using a QIAamp MinElute Virus Spin Kit (Qiagen, Valencia, CA, USA), according to the manufacturer's instructions, with the following modifications: to each 1 ml sample of serum, 3 *μ*g tRNA (Sigma, Stockholm, Sweden), 125 *μ*l kit proteinase and 1 ml kit lysis buffer were added. After a 1-h incubation, 1250 *μ*l of 100% (v/v) ethanol was added. Each sample was filtered through the MinElute column in aliquots until the sample was exhausted. Following standard kit wash procedures (as per the manufacturer's instructions), the DNA was eluted twice in 50 *μ*l elution buffer.

### Germ line DNA extraction

A 9-ml blood sample was collected in a polypropylene tube containing EDTA and gently inverted for a minimum of five times to mix the sample thoroughly. Genomic DNA was extracted using the Nucleon DNA extraction procedure (Tepnel Life Sciences, Manchester, UK).

### Tumour DNA extraction

For analysis of tumour samples, haemotoxylin- and eosin-stained slides were reviewed by a pathologist to ensure sufficient viable tumour (as defined by the presence of at least 100 melanoma cells). DNA was extracted from 40-*μ*m unstained sections of FFPE samples by digesting the samples in proteinase K (Fluka, Buchs, Switzerland) for 48 h, boiling in 5% chelex (Sigma-Aldrich, Dorset, UK) and phase extracting through chloroform-and-ethanol precipitation (Coombs *et al*, 1999). The DNA was re-suspended by adding 50 *μ*l of 0.1 M TE buffer and was then stored at –20°C until use.

### Mutation detection

*BRAF* mutations were detected using a new Amplification Refractory Mutation System (ARMS) allele-specific PCR with Taqman probe assay designed at AstraZeneca (Cheshire, UK) using in-house software (Newton *et al*, 1989). The primer and probe sequences used are shown in Table 1. ARMS primers were designed to detect mutations at amino-acid 600 within the *BRAF* gene. The assay can detect p.V600E, p.V600K and p.V600D mutations within the *BRAF* gene, but does not distinguish between them. Control primers were designed to amplify an area of the *BRAF* gene with no known mutations or single-nucleotide polymorphisms. Primer and probe sequences were modified for the analysis of cfDNA to allow amplification of smaller PCR products.

Each reaction was carried out in a 25-*μ*l reaction volume containing 1 × Brilliant II PCR mix (Stratagene, Cedar Creek, TX, USA), 2 *μ*M each of BRAF ARMS primer and reverse primer, 0.5 *μ*m BRAF probe, 0.1 *μ*m each of control forward and reverse primers, 0.2 *μ*M control probe and 0.8 mg ml^−1^ bovine serum albumin. A 5-*μ*l aliquot of DNA template was added to each reaction. The reactions were amplified on a Stratagene Mx3000P under the following conditions: 95°C for 10 min, followed by 50 cycles of 94°C for 45 s, 60°C for 1 min and 72°C for 45 s. In all cases, samples were assessed in duplicate.

Data were interpreted as follows: if only the control reaction occurred with no mutant reaction, the sample was classified as wild type; if neither reaction occurred, then the sample was classified as unknown, as the concentration of DNA was below the limit of detection; if the mutant reaction occurred, the sample was classified as mutant only if the reaction Δ*C*_t_ between control and mutant reaction was smaller than the Δ*C*_t_ for each of the control wild-type standards on the run to ensure that the mutant reaction was not simply a nonspecific signal (where Δ*C*_t_ is defined as the difference between threshold cycles (mutation *C*_t_−control *C*_t_)). If there was discordance between the replicates or if the Δ*C*_t_ was within 1 *C*_t_ of the Δ*C*_t_ cutoff, then the experiment for the sample was repeated in triplicate, and the sample was considered positive only if all three replicates were positive.

Positive cell line controls were created using DNA extracted from the HT29 cell line, known to be heterozygous for the p.V600E mutation. Human genomic DNA (Roche, Basel, Switzerland) was used as a non-mutant-DNA-containing negative control and appropriate reagent control was used in all PCR runs.

All FFPE-extracted DNA samples found to be positive for the *BRAF* mutant by ARMS were sequenced to determine the exact nucleic acid change.

### Sequencing reactions for tumour DNA

Tumour DNA was added to duplicate PCR assays containing primers that amplified *BRAF* exon 15 (forward primer: 5′-TTTCCTTTACTTACTACACCTC-3′; reverse primer: 5′-CTTTCTAGTAACTCAGCAGCATC-3′). The resulting PCR products were sequenced in forward and reverse directions using ABI BigDye sequencing and analysed using SeqScape (Foster City, CA, USA). A mutation result was accepted if it was present in both forward and reverse sequencing traces, and in duplicate PCRs (to eliminate false-positive mutations occurring because of sample fixation artefacts).

### Cloning and sequencing for *BRAF* mutations

To confirm the presence of *BRAF* mutations in cfDNA from samples in which cfDNA was *BRAF*+ but the matched tumour sample was negative for a mutation by ARMS, cfDNA was extracted from 1 ml of serum and cloned and sequenced for the presence of *BRAF* mutations. Cloning was performed using the TOPO TA Cloning kit (Invitrogen, Paisley, UK) (containing pCR 2.1-TOPO) with chemically competent *Escherichia coli* strain TOP 10F’ (Invitrogen, Paisley, UK). PCR products containing the *BRAF* sequence were obtained using the same primer sequences and conditions as those used for exon 15 *BRAF* sequencing as described above. A mutation result was accepted if it was present in both forward and reverse sequencing traces.

### Reproducibility of *BRAF* detection in cfDNA over 1 year

The reproducibility of *BRAF* detection in cfDNA was tested in 24 serum samples stored at –80°C for 6 months, 13 of which were positive for *BRAF* mutations on initial sampling. A separate set of 24 serum samples stored at −80°C for 12 months were re-tested for *BRAF* mutations, 17 of which were positive for *BRAF* mutations on initial sampling.

The reproducibility of *BRAF* detection in cfDNA stored at −20°C for 6 months was tested on 26 samples, 17 of which had tested positive for *BRAF* mutations at the initial analysis. The reproducibility of *BRAF* detection in cfDNA stored at −20°C for 12 months was tested on a further set of 24 samples, 16 of which had tested positive for *BRAF* mutations at the initial analysis.

### Biostatistical analysis

The primary end point in study D1532C00003 was PFS and, similar to the primary analysis for this study, a multivariate analysis of PFS was carried out for those patients with serum results, using the Cox proportional hazards model allowing for the effect of treatment and adjusting for the following covariates: lactate dehydrogenase (LDH; ⩾2 × upper limit of normal (ULN) *vs* <2 × ULN); *BRAF* mutational status by cfDNA; World Health Organization (WHO) performance status (WHO PS; 0 *vs* 1, 2) and tumour type (uveal *vs* non-uveal).

To assess whether allowing cfDNA-detected *BRAF*+ patients into a selected trial would result in the study population being enriched for patients with differing prognoses from the main study population, analyses were carried out using patients who were *BRAF*+ by tumour but with serum results also available. A univariate analysis was carried out to compare PFS between patients who were *BRAF*+ in serum and patients in whom *BRAF* mutations were not detected. In addition, summary 2 × 2 tables were produced to assess a potential correlation between *BRAF*+, as detected by cfDNA, and the known prognostic factor LDH. LDH levels were available for 190 of the 200 patients enrolled into the study.

## Results

### Assessment of BRAF assay sensitivity

Using the cell line HT29 (known to be heterozygous for the p.V600E mutation), several serial dilution studies of HT29 DNA in human genomic DNA were performed to determine the sensitivity of the BRAF ARMS assay. The *BRAF* mutant could be detected at a level as low as 5 copies of HT29 DNA in a background of 5000 copies of wild-type DNA (0.1% sensitivity, data not shown).

### *BRAF* p.V600 mutation detection in clinical samples

Of the 200 patients enrolled in the trial, 176 tumour samples were obtained; 163 samples were FFPE and the remaining 13 were fresh frozen specimens. Of the 176 tumour samples analysed, 158 (90%) generated acceptable ARMS results. DNA sequence data for *BRAF* were obtained for 147 (84%) tumour samples. In total, 70 *BRAF* mutations in tumour DNA were identified by ARMS (70 of 158 (44%)). Of the *BRAF* mutations detected by ARMS, five were determined by sequencing to be complex g.1798-1799GT>AA changes resulting in a *BRAF* p.V600K alteration, rather than the more common p.V600E (g.1799T>A). Sequencing detected two samples with additional mutation types that could not be captured using the specific ARMS assays in this study: *BRAF* g.1742A>T (p.N581S) and g.1801A>G (p.K601E). Eighteen mutations were detected by ARMS but failed DNA sequencing because of low DNA yields, indicating that ARMS is the more robust method, particularly for analysis of DNA extracted from FFPE specimens; although confined to detecting known mutations.

Of the 96 tumour samples available from patients with cfDNA data, 45 (47%) were detected to be *BRAF*+ by ARMS. Sequencing had confirmed these mutations to be p.V600E in 42 cases and p.V600K in 3 cases. A further tumour sample was shown to harbour a p.K601E mutation, which was not detectable by the ARMS assay design.

Serum samples were available for 126 of the 200 patients enrolled in study D1532C00003; cfDNA was extracted from samples as described and analysed for the presence of a *BRAF* mutation (results summarised in Table 2).

In total, 33 (26%) *BRAF* mutations were detected in cfDNA by ARMS. Of the 126 patients with serum samples, 96 had matched tumour data available. For the remaining 32 patients, tumour data were unavailable either because there was no available tumour sample (*n*=20) or because analysis had failed because of insufficient DNA extracted from the tumour sample (*n*=10).

Five cfDNA samples were positive for a *BRAF* mutation in which no tumour data were available. Of the *BRAF*+ tumours, 25 out of 45 (56%) had *BRAF* mutations detected in the serum. In three samples, *BRAF* mutations were identified in the serum but the tumour was *BRAF* mutation negative. For each of these samples, cfDNA was extracted from a further 1 ml of serum for repeat analysis; in all samples, a *BRAF* mutation was confirmed. In two of these cases, sufficient serum was available to allow extraction of cfDNA from a further 1 ml of serum, and the resultant cfDNA was cloned and sequenced for the presence of *BRAF* mutations. In both these cases, *BRAF* mutations were confirmed in these samples with 13 and 7% of clones positive for a mutation.

In total, of the 96 cases with matched tumour and cfDNA data, the concordance in *BRAF* mutation detection was 76% (95% confidence interval 66–84%). If a *BRAF* mutation was present in tumour DNA, the pick up rate in cfDNA was 56% (95% confidence interval 40–70%).

Importantly, in all samples, analysis of germ line DNA by ARMS was negative for *BRAF* mutations, confirming that any *BRAF* mutations detected were tumour derived.

### Reproducibility

The reproducibility of *BRAF* detection in cfDNA was tested in 24 serum samples stored at −80°C for 6 months and a further 24 serum samples stored at −80°C for 12 months. All serum samples analysed after 6 months storage yielded *BRAF* mutation results identical to the initial analysis. After storage for 12 months, 21 of the 24 serum samples yielded *BRAF* mutation results identical to the initial analysis. In two samples, the *BRAF* mutation was no longer detected and, in one sample, a *BRAF* mutation was detected when initial analysis had been negative. In all of these cases, the tumour sample had been positive for a *BRAF* mutation.

The reproducibility of *BRAF* detection in cfDNA stored at −20°C for 6 months was tested on 26 samples, 17 of which had tested positive for *BRAF* mutations at the initial analysis. At repeat analysis, 16 of the 17 samples that had previously been found to be positive were still *BRAF*+. The one negative sample had previously been positive with a high Δ*C*_t_, suggesting low levels of *BRAF* mutations within the sample. This patient was known to have a *BRAF*+ tumour. A further sample tested positive for a *BRAF* mutation when previously it had tested negative. Again, the Δ*C*_t_ of this sample was high, suggesting low levels of mutant *BRAF* within the sample.

A similar result was observed after analysis of 24 DNA samples stored for 12 months at −20°C. Of the 16 samples previously *BRAF*+, all were *BRAF*+ after 12 months. A further sample was positive for a *BRAF* mutation in which initial analysis had been negative with a high Δ*C*_t_; this sample was from a patient known to have a *BRAF*+ tumour. These data imply that in some samples the level of *BRAF* mutations is very low and sampling differences during analysis could explain the discordant results.

### cfDNA as a prognostic indicator

**Figure 2:**
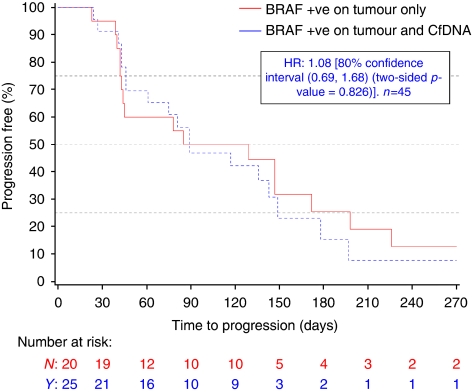
The presence of circulating *BRAF* mutations does not effect progression-free survival. There was no difference in PFS between patients in whom *BRAF* mutations could be detected in the serum compared with those patients in whom cfDNA *BRAF* mutations were not detected. HR calculated using an unadjusted Cox proportional hazards model. Abbreviations: *BRAF*+, *BRAF* mutation positive; cfDNA, cell-free DNA; HR, hazard ratio; CI, confidence interval.

The PFS of the 126 patients with cfDNA results did not differ significantly from the PFS of the study D1532C00003 population as a whole (data not shown). *BRAF* status by tumour sample (in the primary analysis of 200 patients) or cfDNA (*n*=126) was not shown to be a prognostic factor for PFS (data not shown). In addition, in those patients with *BRAF*+ tumours, in whom serum was available for analysis, there was no difference in PFS between patients in whom *BRAF* mutations could be detected in the serum compared with those patients in whom cfDNA *BRAF* mutations were not detected (*n*=45, HR 1.08, 80% confidence interval 0.69, 1.68, two-sided p value=0.826, Figure 2). This suggests that the presence of detectable mutant *BRAF* in serum of patients with *BRAF*+ tumours is not associated with a poorer prognosis on the basis of PFS compared with patients with *BRAF*+ tumours in whom *BRAF* mutations are not detected in cfDNA .

LDH levels were available for 190 patients enrolled in study D1532C00003. Consistent with previous literature, ULN had a worse prognosis on PFS for patients in the study with an LDH level greater than 2x than it did for patients with lower LDH levels. The proportion of patients with *BRAF* mutations in the serum was greater in the elevated LDH group compared with that of patients in the study as a whole (24 *vs* 16%; Table 3) However, if patients with high LDH are excluded from future trials, preselecting on cfDNA *BRAF* serum status will not enrich for poor prognosis patients (as defined by LDH> 2 × ULN).

## Discussion

This study has demonstrated the detection of *BRAF* mutations in cfDNA extracted from the serum of patients with advanced melanoma enrolled in a phase II study of AZD6244 versus temozolomide. The concordance rate of cfDNA *BRAF* mutations with tumour *BRAF* mutations was 56%, which is consistent with that of other reports (Daniotti *et al*, 2007; Yancovitz *et al*, 2007).

Although other groups have demonstrated the feasibility of detecting *BRAF* mutations in serum and plasma of patients with melanoma (Daniotti *et al*, 2007; Shinozaki *et al*, 2007; Yancovitz *et al*, 2007), this is the first study that compares tumour and cfDNA results from a large cohort of patients and demonstrates the potential clinical application of cfDNA mutation detection for patient selection within clinical trials.

Yancovitz *et al* demonstrated *BRAF* mutations in cfDNA extracted from plasma of 14 of 26 (54%) stage IV melanoma patients (Yancovitz *et al*, 2007). Of 17 available tissue samples, the concordance of results was 10 of 17 (59%). Daniotti *et al* compared cfDNA and tumour *BRAF* mutations in 20 patients and found that cfDNA was positive for a *BRAF* mutation in 5 of 13 cases in which the tumour harboured a *BRAF* mutation (Daniotti *et al*, 2007). Shinozaki *et al* demonstrated *BRAF* mutations in 38 of 103 (37%) patients with melanoma (Shinozaki *et al*, 2007). However, they do not record any tumour data to assess concordance of their assay.

Our series identified three cases in which cfDNA was positive for a *BRAF* mutation but the tumour DNA was negative. Yancovitz *et al* and Daniotti *et al* both identified two patients in whom *BRAF* mutations were detected in cfDNA but not in tumour DNA (Daniotti *et al*, 2007; Yancovitz *et al*, 2007). Often, tumour *BRAF* status is derived from a primary lesion that occurred months or years earlier. In our study, the source of tumour material, whether primary or metastatic, was not captured. It is possible that the *BRAF* status of metastatic tumour is different compared with that of primary tumour. However, *BRAF* mutations are thought to develop early in the pathogenesis of melanomas, and analyses of a series of paired primary and metastatic lesions from the same patients indicate that *BRAF* mutations are preserved in metastases (Omholt *et al*, 2003). Heterogeneity in *BRAF* mutation status between metastatic sites may exist (Chang *et al*, 2004), and thus cfDNA might provide a more accurate representation of *BRAF* mutation status within a patient than a biopsy of any single lesion. In this regard, three samples in this study had *BRAF* mutations detected in the serum, wherein the tumour was *BRAF* mutation negative. In two of these samples, the DNA yield from the tumour sample was very low, and in the third sample, histological evaluation of the tumour sample revealed only small amounts of melanoma. Therefore, the difference in mutation results between tumour and cfDNA in these cases may be explained by the fact that the tumour DNA for these samples was not representative of the tumour as a whole. This again highlights the technical challenges in mutation detection in tumour DNA. In two of these three cases, there was sufficient remaining sample to be able to confirm the presence of *BRAF* mutations in cfDNA by cloning and sequencing. These data increase confidence that the *BRAF* mutation was indeed present in cfDNA and that the tumour results are either false negative due to sampling error or not reflective of the mutation status of the metastatic disease.

When considering the use of cfDNA mutation detection as an inclusion criterion for clinical trials, we needed to ascertain whether there was a different overall outcome in those patients with mutant cfDNA compared with those patients with tumour mutations but no cfDNA mutations. If this were the case, then enrolling patients on the basis of cfDNA results may enrich trials for patients with a differing prognosis. Our series has demonstrated that the prognosis by PFS of patients with *BRAF*+ tumours, in whom *BRAF* mutations can be detected in cfDNA, is not significantly different from that of patients with *BRAF*+ tumours in whom *BRAF* mutations cannot be detected in cfDNA. This increases our confidence that enrolling patients into clinical trials on the basis of cfDNA mutation results will not enrich our trial populations for cohorts of patients with an inherently worse prognosis.

Although this study has provided meaningful and interesting results, there are important limitations to this work that require acknowledgement and discussion. First, 191 of the 200 patients enrolled in study D1532C00003 had stage IV melanoma. It is suggested that the detection of cfDNA mutations is tumour-stage dependent, with decreased accuracy in earlier-stage patients. Daniotti *et al* reported *BRAF-*positive cfDNA results in 3 of 13 stage IV melanoma patients but in none of 4 stage I/II patients (Daniotti *et al*, 2007). This ‘stage-dependency’ may restrict cfDNA as a detection method in trials of agents for advanced disease, although more sensitive technologies may alter this in the future.

Second, it should be noted that in the D1532C00003 trial, cfDNA analysis was conducted using serum, which contains high levels of contaminating wild-type DNA sequences after white cell lyses in the clotting process, which may interfere with mutation detection sensitivity. The alternative, plasma, which is processed from blood collected in a tube containing an anticoagulant, contains fewer wild-type DNA sequences and is hence a more sensitive medium for cfDNA mutation detection (Daniotti *et al*, 2007). The use of serum in our study could have reduced the mutation detection rate in cfDNA and our future trials will collect plasma for this purpose.

Third, concern has been raised with regard to the reliability of cfDNA mutation detection in stored blood samples (Sozzi *et al*, 2005; Daniotti *et al*, 2007). Previous reports have demonstrated that cfDNA mutations are no longer detectable in previously positive serum and DNA samples after 12 months of storage (Daniotti *et al*, 2007). In this series, mutation detection was not found to be significantly affected by 6 months of storage of serum samples. However, after 12 months of storage of serum at −80°C, there were three discordant results. Discordant results were also noted when cfDNA extracted from serum was stored at –20°C for 6 or 12 months. In all of these cases, Δ*C*_t_ for the mutation assay was high, suggesting that there was a low proportion of mutant DNA within those samples. This might explain the discordant results; simple sampling differences at the time of the *BRAF* assay may have affected detection. In this series, insufficient sample was available for extensive repeat testing, but these results raise some concern regarding the reliability of mutation detection in cfDNA with low levels of mutant DNA in the sample, especially in stored samples. Future studies will need to focus on repeated testing of initial samples to fully establish the reliability and precision of these tests. The use of plasma or more-sensitive technologies in future trials may reduce the number of samples with discordant results on repeated testing.

Finally, and importantly, not all patients with *BRAF*+ tumours had *BRAF*+ cfDNA. As discussed, it is possible that there are some differences in the mutation status between primary tumour and metastatic deposit, but the most likely explanations are that either the tumour cfDNA is shed at such a low level that it is below the sensitivity of the assay or there are true biological differences between tumours in different patients, indicating that cfDNA is shed at a higher level in some patients than in others. Recent reports from a study of cfDNA mutations in patients with operable colorectal cancer using an ultra-sensitive technology (BEAMing) suggest that mutated cfDNA fragments are present at levels as low as 0.18% (Diehl *et al*, 2008). Development of these increasingly sensitive technologies may improve detection rates in the future.

Although the exact source and nature of cfDNA remain unclear, the potential applications of its use are becoming increasingly apparent. It offers significant advantages over tumour DNA analysis: it is less invasive than tissue biopsies and results are quicker to obtain (particularly within clinical trial scenarios when tissue samples are often spread across many sites and countries). In addition, cfDNA can provide us with the opportunity to detect the current or real-time mutation status of a tumour and ultimately could lead to serial sampling to assess tumour progression or the development of resistant mutations. This study has demonstrated that cfDNA analysis could provide an opportunity for the detection of tumour-specific mutations in patients with advanced melanoma, allowing for speedy access to novel agents and enrolment into clinical studies without waiting for tissue procurement. This technology provides patients who have no available tissue samples with the opportunity to be considered for a study without having to undergo further invasive procedures to obtain tissue samples. The study has demonstrated that the detection of *BRAF* mutations in cfDNA is not significantly prognostic in advanced melanoma, and that, provided high LDH patients are excluded from the study population, entering patients by cfDNA analysis into a *BRAF*+-selected trial will not enrich for a poor prognostic study population. Further studies on AZD6244 and other targeted agents will focus on improving the tissue/cfDNA concordance rate and will aim to further validate cfDNA as a surrogate marker for tumour DNA mutations and as an inclusion criterion for clinical studies.

## Figures and Tables

**Figure 1:**
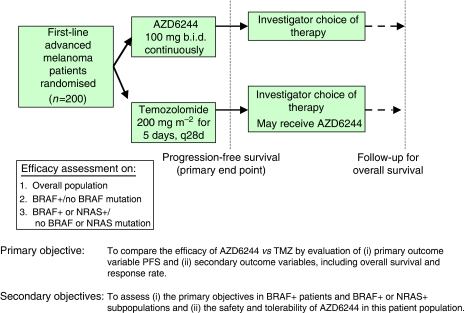
Melanoma phase II study design. Abbreviations: *BRAF*+, *BRAF* mutation positive; NRAS+, NRAS mutation positive; PFS, progression-free survival; TMZ, temozolomide.

**Table 1:** Primer and probe sequences

**ARMS assay**	**ARMS primer (5′-3′)**	**Common primer (5′-3′)**	**TaqMan probe (5′-3′)**	**Size (bp)**
*BRAF* for FFPE 1799T>A	AAAAATAGGTGATTTTGGTCTA GCTACATA	TAGTTGAGACCTTCAATGACTTT CTAGTAA	Yakima Yellow –AATCTCGATGGAGT GGGTCCCATCAGTTTGAACA-Bhq	179
Control for FFPE	AGGACACCGAGGAAGAG GACTT	GGAATCACCTTCTGTCTTCATTT	Cy – CCATCTTCTTCCTGCCTGATGA GGGGAAA-Elle	252
*BRAF* for cfDNA	AAAAATAGGTGATTTTGGTCTA GCTACATA	CATCCACAAAATGGATCC AGACAA	Yakima Yellow – GATGGA+GTGGGTC+ CCATC+AG-Bhq	91
Control for cfDNA	CTCCAGATCTCAGTAAGG TACGG	GGGAAAGAGTGGTCTCTCATC	Cy5 – CATGA+AG+AGATTAAT GGCAG+AGTG+CC-Elle	101

Abbreviations: ARMS=Amplification Refractory Mutation System; cfDNA=circulating free DNA; FFPE=formalin-fixed paraffin embedded tissues.

**Table 2:** Summary of *BRAF* mutation analysis of tumour and cfDNA in *n*=126 patients

		**Tumour DNA**			
		**BRAF positive**	**BRAF negative**	**BRAF unknown**	**Total**
cfDNA	*BRAF* positive	25	3	5	33
	*BRAF* negative	20	46	27	93
Total		45	49	32	126

Abbreviation: cfDNA=circulating free DNA.

**Table 3:** Correlation of *BRAF* mutations in cfDNA with poor prognostic factors (lactate dehydrogenase)

	**CfDNA patients (*n*=126); serum mutation results**		**Total study**
**LDH**	**BRAF positive (*n*=33)**	**BRAF negative (*n*=93)**	**population (*n*=200)**
LDH<2XULN	24 (73%)	82 (88%)	158 (79%)
LDH=2 × ULN	8 (24%)	8 (9%)	32 (16%)
LDH unknown	1 (3%)	3 (3%)	10 (5%)

Abbreviations: cfDNA=circulating free DNA; LDH=lactate dehydrogenase; pts=patients; ULN=upper limit of normal.
